# Early psychiatric morbidity in a Brazilian sample of acute ischemic stroke patients

**DOI:** 10.6061/clinics/2018/e055

**Published:** 2018-04-21

**Authors:** Vinicius S.P. Pedroso, Andre R. Brunoni, Érica L.M. Vieira, Ricardo E. Jorge, Edward C. Lauterbach, Antonio L. Teixeira

**Affiliations:** I Divisao de Neurociencias, Laboratorio de Investigacao Medica Interdisciplinar, Faculdade de Medicina, Universidade Federal de Minas Gerais (UFMG), Belo Horizonte, MG, BR; II Programa de Graduacao em Neurociencia, Instituto de Ciencias Biologicas, Universidade Federal de Minas Gerais (UFMG), Belo Horizonte, MG, BR; III Departamento de Psiquiatria, Faculdade de Medicina (FMUSP), Universidade de Sao Paulo, Sao Paulo, SP, BR; IV Menninger Department of Psychiatry & Behavioral Sciences, Baylor College of Medicine, Houston, TX; V Department of Psychiatry and Behavioral Sciences and Department of Internal Medicine (Neurology Section), Mercer University School of Medicine, Macon, GA; VI Neuropsychiatry Program, Department of Psychiatry and Behavioral Sciences, McGovern Medical School, University of Texas Health Science Center at Houston, Houston, TX

**Keywords:** Stroke, Neuropsychiatry, Depression, Anxiety

## Abstract

**OBJECTIVE:**

Stroke is a major public health problem worldwide, and its neuropsychiatric sequelae are frequent and disabling. Furthermore, there is evidence that these sequelae impair recovery. Brazil has the highest stroke rates in Latin America, but data on the frequency of neuropsychiatric disorders in these patients are scarce. This study aimed to identify mental disorders among in-hospital patients with acute ischemic stroke.

**METHODS:**

The Mini International Neuropsychiatric Interview-Plus (MINI-Plus) was applied to 60 patients during the first week of hospitalization.

**RESULTS:**

Psychiatric disorders were diagnosed in 55% of the patients. A wide range of neuropsychiatric disorders have been identified, mainly mood and anxiety disorders. Specifically, we identified major depression (26.7%), alcohol abuse or dependence (11.7%), specific phobia (8.3%), generalized anxiety disorder (6.7%), psychosis (5.0%), social phobia (3.3%), adjustment disorder (3.3%) and panic disorder (1.7%).

**CONCLUSION:**

Psychiatric comorbidity should be evaluated as part of the rehabilitation of stroke patients and should be carefully examined by physicians.

## INTRODUCTION

Stroke is a leading cause of disability and death worldwide 1. Compared to data for the United States (US), where epidemiological statistics show that 610,000 new cases occur each year, epidemiological data are relatively scarce in Brazil. Still, the available information shows that stroke is the major cause of death in Brazil, accounting for approximately 100,000 deaths annually 2,3.

However, over the last few decades, there has been a global trend of decreased mortality from stroke. This decrease is probably due to the improvement in stroke treatment and to preventive measures, such as the treatment of hypertension. In Brazil, decreases in the death rates from stroke are also evident, but these decreases are restricted to the south and southeastern regions 2,3. Currently, an estimated 5 million stroke survivors live in the US. In Brazil, which has the highest stroke death rates in Latin America, the number of stroke survivors is estimated to be at least half that in the US. As acute stroke management continues to improve, the number of survivors with varying degrees of disability will increase even further. Since stroke often results in major changes in the life of the patient, factors associated with morbidity have received increasing attention 2,3.

A multiplicity of behavioral and affective changes can be associated with vascular lesions of the central nervous system, with the possibility of acute damage to circuits associated with the processing of emotions and cognition. Stroke is often associated with psychiatric symptoms, such as anxiety, depressed mood and apathy 4. Behavioral complications of stroke, although recognized for over a century, have never received the attention devoted to other stroke consequences, such as motor impairment, language disorders, and cognitive deficits. However, beyond the impacts that psychiatric complications have on neurological recovery, psychiatric complications have a significant influence on the professional lives and interpersonal relationships of the patients, on their families and on their caregivers, by modifying patient autonomy, self-esteem and quality of life (QOL) in general 5,6.

Despite such importance, data in the Brazilian literature on the profile of neuropsychiatric disorders in patients after acute stroke are limited. Therefore, the aim of this study was to evaluate the occurrence of psychiatric disorders in Brazilian patients affected by acute ischemic stroke.

## MATERIALS AND METHODS

This was a cross-sectional study. Patients with a diagnosis of acute ischemic stroke admitted to the Stroke Unit of the Hospital Municipal Odilon Behrens, Belo Horizonte, Brazil, underwent neuropsychiatric evaluations. We included consecutively encountered patients of both genders who were older than 45 years of age (to exclude stroke in young adults, in whom stroke could be associated with diverse etiologies, such as autoimmune diseases) and who provided written informed consent. Individuals with hemorrhagic stroke, autoimmune diseases, active infectious diseases, recent acute myocardial infarction, decreased level of consciousness (Glasgow Coma Scale <15), clinical history of dementia, or severe aphasia or who underwent recent neurosurgery were excluded. Data were collected from November 2013 to November 2015.

In a semi-standardized interview, we collected data on the participant’s sociodemographic characteristics, previous history of cerebrovascular disease, clinical comorbidities, use of thrombolytic therapy, use of medication, neuroimaging results, electrocardiogram and physical examination (height, weight, blood pressure, waist circumference and neurological examination). These variables were used to calculate the Framingham Risk Score for cardiovascular disease 7. Stroke was classified in accordance with the Bamford 8 and TOAST 9 classifications. Stroke severity was quantified by using the National Institutes of Health Stroke Scale 10 (NIHSS), and disability was assessed with the Modified Rankin Scale 11 (mRS) and Functional Independence Measure 12 (FIM). Subsequently, patients were interviewed by a trained psychiatrist, using the Mini International Neuropsychiatric Interview-Plus (MINI-Plus) - Brazilian version 5.0.0 13. The MINI-Plus allows the evaluation of current diagnoses and a previous history of mental disorders. In this study, diagnoses were considered “current” if initiated after the ischemic event, as “past” if there was a history of symptoms before but not after the stroke, and as “past/current” if there was history of symptoms before and after the stroke. Two weeks after the evaluation, patients were contacted by phone to ensure the stability of the diagnosis during that period through a structured interview based on the MINI-Plus.

### Ethics

The study was approved by the local research ethics committee and is in accordance with the Helsinki Declaration of 1975, which was revised in 1983.

## RESULTS

### 1. Demographic characterization of the patients

Sixty patients were enrolled. Table 1 presents the demographic characteristics of the sample. The mean age was 64.3 years. Male participants (70%) of black/brown skin color (87%) predominated. Most patients were living without a partner or spouse (55%). The average educational level was low (5.83 years), which was reflected in a monthly income below three minimum wages in most cases (81.7%). Most of the patients were not actively working (73.3%). Most patients stated some sort of religious support or belief (86.7%).

### 2. General health data

Table 2 shows the general health characteristics of the studied sample. Hypertension and obesity were highly frequent (95% and 60%, respectively), followed by dyslipidemia (36.8%) and diabetes (30%). Smoking was also frequent. Most patients were using antihypertensive drugs (95%), antiplatelet drugs (70%) or statins (65%). Physical assessment yielded a trend toward overweight and high blood pressure. The average Framingham score was 21.7.

### 3. Stroke characteristics

Table 3 shows the characteristics related to stroke in the studied sample. Thrombolytic therapy was used only in 5% of patients. The time elapsed since the ischemic event until our evaluation was 5.4 days on average. All patients underwent computed tomography neuroimaging. According to the TOAST and Bamford classifications, most cases corresponded to lacunar strokes (53.3%), followed by atherothrombotic strokes. The lesion sites were mainly found in the right hemisphere (61.7%), affecting the frontal lobes, the basal ganglia and the internal capsule. The mean NIHSS score at admission was 3.83, and the mean mRS was 2.4. The average FIM score was 113.67.

### 4. MINI-Plus diagnosis

Table 4 shows the results of the assessment using the MINI-Plus. At least one mental disorder was diagnosed in 55% of the cases. Depression was the most frequent disorder, with a frequency of 26.7%, including one case of depression with psychotic symptoms (mood-congruent delusions and auditory hallucinations). A past history of depressive disorders was found in 5% of the patients, but none of them was diagnosed with current major depression in our assessment. As a group, anxiety disorders, especially phobias and generalized anxiety disorder (GAD), were found in 23.3% of the cases. Alcohol-use disorders were also frequent and found in 11.7% of the cases. Psychosis without other specifications was observed in 5% of the cases.

## DISCUSSION

To our knowledge, this is the first study to perform a systematic and comprehensive assessment of the psychiatric profile of Brazilian patients with acute ischemic stroke. A detailed description of the clinical and demographic characteristics of the sample was performed, followed by a structured psychiatric evaluation using a standardized tool for diagnosis. Sixty patients were evaluated.

The analysis of the sociodemographic characteristics of the sample allows us to trace the profile of the patients assisted by a public hospital, which is considered the local reference center for the care of stroke. Thus, we observed that the sample comprised older patients who were predominantly unemployed, had a low income and low education level, and were socially vulnerable.

Patients had a high rate of clinical comorbidities, especially hypertension, obesity, dyslipidemia, diabetes and smoking. A similar medical comorbidity profile was found in a previous study, performed at the same hospital, that investigated the role of Chagas disease as an independent risk factor for the occurrence of stroke 14. The observations of mean BMI values above 25 kg/m^2^ and high mean blood pressure values, despite the use of medication, draws attention to the unsatisfactory clinical management of patients prior to admission. The Framingham score for the sample was calculated from the recorded clinical variables. This score was developed based on information collected in epidemiological population studies performed over 36 years and estimates the probability of stroke from clinical information 7. The mean score observed was 21.27. This value indicates that the probability of stroke in 10 years is over 30% for men and 14% for women, and this value confirms the high-risk profile for the development of stroke in this sample.

Interviews occurred, on average, 5.4 days after the ischemic event, in accordance with the purpose of evaluating patients during the acute period after stroke. Most of the observed individuals presented with lacunar stroke, with a predominance of lesions in the middle cerebral arterial topography of the right hemisphere. The NIHSS, mRS and FIM scales were used to measure the initial severity of the ischemic event and its resulting disability. The mean results were below 5 for the NIHSS and below 3 for the mRS, indicating a mild impact of stroke in the sample. This fact is reflected in the measurement of patient dysfunction obtained by the FIM, a scale that ranges from 18 (worst outcome) to 126 (best outcome) points. The average value, 113.67, found in the sample was compatible with a mild functional deficit. Most likely, the timing of the evaluation and the inclusion/exclusion criteria of the study biased the sample to include mostly minor strokes. This may influence the generalizability of the results.

Despite the mild functional impact, the assessment through a structured psychiatric interview revealed the presence of mental disorders, especially depressive and anxiety disorders, in approximately 55% of cases. In accordance with other studies, depression was the most frequent psychiatric disorder 15,16. Chemerinski and Robinson have observed that the frequency of depression among inpatients during the acute phase of stroke is approximately 22% for major depression and 17% for minor depression 4. In outpatient samples (ranging from 3 months to 10 years post stroke), the frequency is approximately 23% for major depression and 35% for minor depression, while community samples exhibit mean prevalence rates of 13% and 10%, respectively. A meta-analysis showed that the prevalence of depression at any time after stroke was 29%. We found a similar number, namely, a frequency of 26.7% of depression in our sample. In this sense, a systematic review of Brazilian studies that evaluated the prevalence of depression after stroke in different contexts found prevalence rates that varied from 20 to 59% 17.

Anxiety disorders are also common after stroke. Between 25% and 50% of patients develop GAD during the first months after stroke, with a small reduction in incidence within the following three years 18. Burton et al. reported that anxiety disorders affected 20% to 25% of patients at any time after stroke 19. However, most of the studies of patients affected by stroke have not effectively explored the presence of anxiety disorders. Thus, there are scarce data on specific categories, such as panic attacks, agoraphobia or phobias. According to Burton et al., phobic disorders and GAD are the most common types of anxiety disorders after a stroke 19. In line with this observation, we found that the frequency of anxiety disorders, especially phobias and GAD, was 23.3%.

Considering that mental disorders negatively influence the recovery of patients after stroke and that factors associated with social vulnerability are risk factors that complicate treatment, the high frequency of depression and anxiety disorders calls attention to the possible consequences that may result if patients are not properly identified and treated, even when the functional impact of stroke in patients is mild 20,21. The relationship between depression after stroke and functional impairment is intricate. Depressed patients have a significantly greater disability in the activities of daily living (ADLs) than euthymic individuals with equivalent neurological deficits 4. Depression negatively influences patient involvement in rehabilitation programs and is associated with increased institutional care and use of health services. These findings suggest a phenomenon of mutuality in which depression influences the recovery of ADLs, while ADLs impairment affects the severity and duration of depression. Increased mortality may be the most important aspect of depression in the prognosis of stroke. In this sense, depression may be a risk factor for increased death as early as 1 year and as late as 7 years after stroke 22.

As with depression, anxiety disorders may be linked to psychological factors after stroke. In addition to fears of the occurrence of new stroke events, concerns about the possibility of failing to control motor, cognitive and emotional reactions in different environments are common in stroke patients. This may be reflected in a decreased perception of QOL. Indeed, patients with severe stroke and high levels of anxiety present with worse QOL 23.

The decreased QOL and negative functional impact caused by mental disorders can further complicate the management of clinical comorbidities and adherence to treatment, contributing to an increase in the overall risk of complications. Thus, psychiatric comorbidity should be evaluated as part of the rehabilitation of stroke patients and should be carefully examined by physicians.

Finally, the study design, its inclusion and exclusion criteria, the evaluation time (approximately 5 days on average) and the relatively small sample size impose limitations on the generalizability of the data. The manifestations of mental disorders after a stroke can occur in dynamic ways that vary over time, and the telephone evaluation after two weeks was made in order to minimize the problems associated with early evaluation. However, the data obtained allow us to generate an overview of the importance of psychiatric evaluations in this population and provide valuable information for future studies on the subject.

## AUTHOR CONTRIBUTIONS

All six authors contributed sufficiently to this work to take public responsibility for appropriate portions of the content. Pedroso VS, Brunoni AR and Vieira EL contributed to writing the text and to the analysis of the data. Jorge RE and Lauterbach EC contributed to the revision of the text, revision of the English language and analysis of the data. Teixeira AL contributed to the overall review of the work.

## Figures and Tables

**Table 1:** Demographic features of a sample of 60 in-hospital patients after ischemic acute stroke.

Demographic features (n=60)	
Age (years; mean ± standard deviation)	64.3 (±8.79)
Gender	
Male	70.0%
Female	30.0%
Skin color	
Black	31.7%
Brown	55.3%
White	13.0%
Marital status	
Single	3.3%
Married	40.0%
Living together	5.0%
Divorced	26.7%
Widower	25.0%
Schooling (mean ± standard deviation)	5.83 (±3.31)
Monthly income	
Without regular income	6.7%
<3 minimum wages*	75.0%
Between 3 and 10 minimum wages*	18.3%
Current employment status	
Active	26.7%
Unemployed	15.0%
Retired	45.0%
Receiving social assistance	13.3%
Religion	
Catholic	50.0%
Protestant	36.7%
No religion	13.3%

* One minimum wage is the lowest monthly remuneration that employers may legally pay workers in Brazil. Currently, the value is R$ 880,00 (approximately US$ 270.00).

**Table 2:** General health features of a sample of 60 patients after ischemic acute stroke.

General health features (n=60)	
Clinical comorbidities	
Hypertension	95.0%
Diabetes	30.0%
Obesity	60.0%
Dyslipidemia	36.8%
History of myocardial infarction	6.7%
Congestive heart failure	1.7%
Epilepsy	1.7%
Smoking	
Current	26.7%
Previous	18.3%
Medications in use	
Antihypertensive drugs	95.0%
Statins	65.0%
Hypoglycemic drugs	30.0%
Aspirin or Clopidogrel	70.0%
Psychotropic drugs	1.7%
Clinical data (mean ± standard deviation)	
Body mass index	26.4 kg/m2 (±4.49)
Abdominal circumference	97.07 cm (±13.47)
Systolic blood pressure	141.83 mmHg (±20.38)
Diastolic blood pressure	88.83 mmHg (±11.21)
Framingham score (mean ± standard deviation)	21.7 (±3.28)

**Table 3:** Features of acute ischemic stroke in a sample of 60 patients.

Stroke features (n=60)	
Use of thrombolytic therapy	5.0%
Elapsed time since stroke (mean ± standard deviation)	5.4 days (±1.53)
TOAST classification	
Cardioembolism	8.3%
Large-artery atherosclerosis	38.4%
Small vessel occlusion	53.3%
Bamford classification	
LACS	53.3%
PACS	38.4%
POCS	8.3%
Affected hemisphere	
Right	61.7%
Left	38.3%
Lesion site	
Frontal lobe	30.0%
Temporal lobe	6.7%
Occipital lobe	8.3%
Parietal lobe	0.0%
Basal ganglia	28.3%
Internal capsule	35.0%
Cerebellum	1.7%
NIHSS	3.83 (±3.06)
Modified Rankin scale (at admission)	2.4 (±0.97)
Functional independence measure	113.67 (±12.44)

LACS: Lacunar stroke; PACS: Partial anterior circulation stroke; POCS; Posterior circulation stroke; NIHSS: National Institutes of Health Stroke Scale.

**Table 4:** Psychiatric diagnosis established by the use of MINI-Plus in a sample of 60 patients after ischemic acute stroke.

Diagnosis by MINI-Plus (n=60)	
MINI-Plus	
Major depression (c)	26.7%
Without psychotic symptoms	25.0%
With psychotic symptoms	1.7%
Depression (p)	5.0%
Anxiety disorders	23.3%
Social phobia (p/c)	3.3%
Specific phobia (p/c)	8.3%
Generalized anxiety disorder (p/c)	6.7%
Adjustment disorder (c)	3.3%
Panic disorder (p/c)	1.7%
Psychosis (not otherwise specified) (c)	5.0%
Alcohol dependence/abuse (p/c)	11.7%
No mental disorder (p/c)	45.0%

c: Current; p: Past; p/c: Past and Current.
